# ID 42 - Painel Interno de Produtos em Avaliação de Tecnologias em Saúde Desenvolvidos por Secretarias Estaduais de Saúde: uma ferramenta para intercâmbio de experiências

**DOI:** 10.5327/2237-9622.2025.v34s1.42

**Published:** 2025-11-25

**Authors:** Rodrigo Pereira de Almeida, Bruna Marmett, Ana Paula Blankenheim, Bárbara Cristiane da Silva, Roseana Boek Carvalho, Marina Petrasi Guahnon, Muriel Primon de Barros, Gilson Pires Dorneles, Suena Medeiros Parahiba, Maicon Falavigna

**Keywords:** painel de produtos, Avaliação de Tecnologias em Saúde, Secretarias Estaduais de Saúde

## Abstract

**Introdução:**

A Avaliação de Tecnologias em Saúde (ATS) é um tema emergente no contexto das Secretarias Estaduais de Saúde (SES), onde os processos e os produtos de ATS ainda são incipientes para apoiar a tomada de decisão em nível local. As demandas desenvolvidas permanecem majoritariamente restritas a questões internas, carecendo de disseminação e maior colaboração entre as SES. Assim, uma ferramenta para o registro de informações sobre produtos desenvolvidos ou em desenvolvimento possui potencial no fortalecimento da colaboração em ATS no contexto das SES, uma vez que várias compartilham de necessidades semelhantes. O objetivo deste resumo foi descrever o processo de desenvolvimento de um painel interno de produtos em ATS elaborados pelas SES.

**Método:**

O desenvolvimento da ferramenta foi realizado em quatro etapas: 1) criação de um formulário para coleta de dados; 2) desenvolvimento de um painel interativo; 3) teste da ferramenta, e; 4) validação da ferramenta. Na primeira etapa, um formulário no Google Forms foi estruturado para obter dados de identificação dos respondentes e informações sobre os produtos de ATS desenvolvidos pelas SES. A segunda etapa consistiu na estruturação da apresentação dos dados coletadas pelo formulário em um painel interativo, utilizando o Google Looker. Esse painel é composto por uma planilha e um sistema de filtros que permitem aos usuários identificarem as principais informações dos produtos. A etapa de testes foi realizada por meio do preenchimento do formulário com demandas hipotéticas, a fim de verificar sua fluidez e observar a estruturação dos dados no painel. Por fim, foi realizada a validação da ferramenta por um profissional com expertise na área de ATS para contribuições na estruturação da ferramenta.

**Resultados:**

Na etapa 1, o formulário foi estruturado em duas seções: a identificação do respondente, e descrição e informações dos produtos desenvolvidos. Nesta segunda seção, foram abordadas informações como: a) a SES responsável pela produção, as datas previstas de início e término da elaboração; b) o tipo de documento desenvolvido; c) características da demanda, como o nome e a indicação clínica da tecnologia avaliada, a área médica, o motivo da demanda, o demandante e o status da produção. Definiu-se que só seriam publicizadas as informações referentes aos produtos, mantendo o anonimato do respondedor. Na etapa 2, as respostas referentes aos produtos foram exportadas a uma planilha gerada automaticamente e vinculadas ao Google Looker, resultando em um painel interativo que exibe as informações de cada um. Nas etapas 3 e 4, após os testes e a validação, foram realizados ajustes breves para facilitar o preenchimento do formulário e visualização do painel. A disseminação e a difusão dessa ferramenta ocorrerão pela disponibilização para os membros das 27 SES que atuam diretamente com ATS. A versão preliminar do painel desenvolvido está disponível no seguinte endereço: https://lookerstudio.google.com/reporting/28d65bd3-eb8a-4573-b5f2-642d3556c9e1.

**Conclusão:**

Esta ferramenta vai permitir a compilação dos dados coletados em um painel interativo, de fácil acesso e que se mantém em constante atualização. Além disso, ela pode auxiliar na promoção de ATS e fomentar a interação e a colaboração entre os profissionais envolvidos nesta área nas SES, facilitando o compartilhamento de informações e o desenvolvimento de trabalhos colaborativos.

